# Pathogens and Disease Play Havoc on the Host Epiproteome—The “First Line of Response” Role for Proteomic Changes Influenced by Disorder

**DOI:** 10.3390/ijms19030772

**Published:** 2018-03-08

**Authors:** Erik H. A. Rikkerink

**Affiliations:** The New Zealand Institute for Plant & Food Research Ltd., 120 Mt. Albert Rd., Private Bag 92169, Auckland 1025, New Zealand; erik.rikkerink@plantandfood.co.nz; Tel.: +64-9-925-7157

**Keywords:** intrinsically disordered proteins, epiproteome, disordered protein platform, molecular recognition feature, post-translational modifications, physiological homeostasis, stress response, RIN4, p53, molecular machines

## Abstract

Organisms face stress from multiple sources simultaneously and require mechanisms to respond to these scenarios if they are to survive in the long term. This overview focuses on a series of key points that illustrate how disorder and post-translational changes can combine to play a critical role in orchestrating the response of organisms to the stress of a changing environment. Increasingly, protein complexes are thought of as dynamic multi-component molecular machines able to adapt through compositional, conformational and/or post-translational modifications to control their largely metabolic outputs. These metabolites then feed into cellular physiological homeostasis or the production of secondary metabolites with novel anti-microbial properties. The control of adaptations to stress operates at multiple levels including the proteome and the dynamic nature of proteomic changes suggests a parallel with the equally dynamic epigenetic changes at the level of nucleic acids. Given their properties, I propose that some disordered protein platforms specifically enable organisms to sense and react rapidly as the first line of response to change. Using examples from the highly dynamic host-pathogen and host-stress response, I illustrate by example how disordered proteins are key to fulfilling the need for multiple levels of integration of response at different time scales to create robust control points.

## 1. Introduction

Survival of both individuals and a species is predicated, in no small measure, on their ability to respond to a changing environment. Faced with the challenge of drastic changes, organisms have stark options of fight or flight. Flight comes with its own series of challenges (including adapting to a new environment, or competing with others that have already occupied the new niche). Either way there is a strong evolutionary imperative to acquire an ability to adapt. Adaptation is likely to require response at different time scales from immediate (at the level of the individual) to geological scale (at the level of species and genus). Rapid response can be both a benefit and a cost, as a quick change in direction can sometimes prove to be detrimental in the fullness of time and might, therefore, be likely to favor rapid responses that are also readily reversible. Critical decision points used by the organism to drive response in a particular direction need to be robustly integrated into the core physiology of the cell. In this review I argue in favor of the broader interpretation of the term epiproteome to encapsulate the concepts that (1) changes at the post-translational level are ideally placed to respond in real time and that (2) flexible proteins displaying significant disorder are ideal platforms that can be decorated with post-translational changes and used to integrate responses that potentially have competing impacts on cellular resources.

The term epiproteome was first coined by Dai and Rasmussen [1] to refer to proteomic changes directly associated with epigenetic modifications, namely histone acetylation. Some researchers argue that histone modifications are part of epigenetics, although others argue that their lack of heritability means they should not be included in that term. A search for epiproteome/epiproteomics in PubMed Central yields references to post-translational modification (PTM) changes in histones and a small number that use the term in a wider sense to refer to other PTM [2]. Below I argue in favor of the broad interpretation that includes all PTM.

I suggest that an understanding of the epiproteome (i.e., changing alternative post-translational protein states) in combination with the critical nodal positions occupied by disordered proteins, provides a new basis to comprehend the hypervariable PTM theatre. Its features enable integration of multiple post-translational signals to match the demands of a flexible response. The best examples of hypervariable theatres of response to stress are the battle between hosts and their pathogens and/or their changing environment. Epiproteomic changes offer the host an elegant real-time control of its responses. Unfortunately, this also makes the PTM theatre an Achilles heel, able to be exploited by pathogens. Arguably this explains why so much of a pathogens weaponry appears to be enzymatic and focused on the PTM level of host organisation [3].

## 2. Review

Below I address five key points or questions that address the key demands on a highly integrated cellular stress control point, namely: (1) A broad interpretation of the concept of the epiproteome; (2) How an organism can communicate between (and marshal) sets of proteins that need to respond to stress while also integrating the, on occasion conflicting, demands of distinct but simultaneous stresses; (3) Are there some key exemplars in plants and animals that point towards solutions to meet the demanding challenge of multiple stresses? (4) What are the characteristics required for a node that can successfully integrate response to simultaneous challenges? (5) How can multiple diverse signals be coordinated in real-time to deliver a coherent response?

### 2.1. Why Use the Broad Interpretation of the Term Epiproteome?

Our concepts of how the molecular machinery in a cell operates have changed radically over the last three decades. One-dimensional models of static proteins acting alone to promote a particular enzymatic step have been superseded by an understanding that proteins typically act as parts of molecular nano-machines in complexes, and are dynamically controlled by a combination of their own intrinsic flexibility [4], their micro-environment, location and their interactions with their partners. We know that robust control of cellular processes needs to occur at multiple levels [5] that can include modifications of chromatin, transcription, post-transcription, translation and PTM. Epigenetic changes and their role in host plasticity have been widely discussed over the last decade [6,7], including their role in responding to challenges such as pathogens [8]. More recently epitranscriptomic changes have become a topic of renewed interest [9]. It is timely therefore to focus on the epiproteome and the key role that PTM could play in coordinated cellular response to pathogens and other stresses. There are of course thousands of papers referring to specific post-translational modifications or similar terms. Initially epiproteomics referred simply to changes in the specific proteome associated with DNA epigenetic changes [1]. And indeed it is still sometimes used in this way now [10]. Only recently have papers used the term to refer to the sum of all post-translational changes in all proteins or a subset such as the redox or cysteine epiproteome [2,11]. The term epiproteomics evokes a parallel with the temporal nature of epigenetics that is entirely appropriate, and perhaps even central, to the importance of the PTM level of control. Therefore below the term epiproteome is applied in a much more general way to any post-translational modification of any protein in any protein complex. PTM can lead to a large ensemble of forms of the components of the proteome existing in a dynamic state within a cell. Unfortunately, the research tools required to analyse PTM states properly are still expensive to run and hence our current picture of the dynamics of these states is inadequate. PTM alternative states are however likely to be more significant than random noise and there are numerous individual cases where this is confirmed.

### 2.2. Marshalling a Diverse Set of Responding Proteins More or Less in Unison

When combined with the concept of the role of intrinsic disorder in signalling, the significance of epiproteomic changes are placed in a new light. Coherently controlled epiproteomic changes would have the potential to alter the response of many proteins simultaneously, whether they be members of the same protein complex or dispersed complexes that need to be coordinated with each other. Individual PTM changes have been shown to play key roles in a number of different properties including the formation or dissolution of protein interactions [12], the conformation of a protein [13], the membrane localisation of a protein [14] or the inactivation [15] or degradation [16] of a protein. When such changes are driven by a significant change in physiology of the entire cell, such as its redox potential or pH, there is significant scope for matching coordinated changes in the protein complexes within the cell that need to react to the new state. Thus, the epiproteome is uniquely positioned to play a vital role in marshalling multi-protein responses. There are some known examples of this in situations of stress in nature already. A plant pathogen effector was recently shown to acetylate several proteins that interact with each other in a complex [17]. Another example is the cell signalling that results from electrophilic oxidized lipid products, so called reactive lipid species (RLS), that can react with the amino acids cysteine, lysine and histidine because of their nucleophilic nature. RLS effects on signalling events are largely restricted to the modification of cysteine residues in proteins. RLS induced modifications appear to participate in multiple physiological processes including inflammation, induction of antioxidants and even cell death through the modification of signalling proteins [18].

### 2.3. Animal and Plant Exemplars: p53 and RIN4

**Figure 1:**
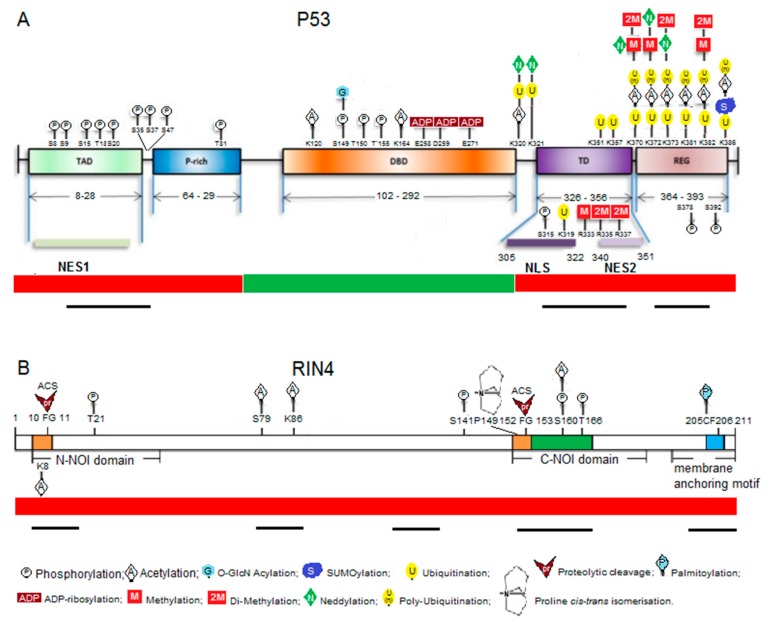
Multiple PTMs cluster in the Molecular Recognition Features within the disordered regions in p53 (**A**) and RIN4 (**B**). Consensus disordered regions are indicated by the red bar at the bottom while ordered regions are indicated by the green bar, putative MoRFs within the disordered regions are indicated by black bars as determined by Uversky (2016; p. 53, [23]) or Sun et al. (2014; RIN4, [32]) by application of disorder prediction programs. PTMs for p53 are a modified form of those identified by Gu and Zhu (2012) [24]. TAD: Transactivation domain; P-rich: proline-rich domain; DBD: DNA binding domain; TD: tetramerization domain; REG: C-terminal regulatory domain; NES1/2: N-terminal (1) and C-terminal (2) nuclear export sequences; NLS: nuclear localization sequence; N-NOI: N-terminal Nitrate induced domain; C-NOI: C-terminal Nitrate induced domain; ACS: AvrRpt2 cleavage site.

The p53 transcription factor in mammals is best known as a target for cancer therapy and understanding the interaction between stress and cancer but is also associated with facets of aging and microbial responses [19]. Additionally, p53 is a target for microbial manipulation by both viruses and bacteria [20]. The p53 protein interacts with a remarkable array of partners and a key characteristic that allows p53 to act as such a key node/hub is believed to be its disordered characteristics [21]. A particularly pertinent facet of disorder in this discussion is its accessibility to PTM events. In highly structured proteins, only a minority of surface exposed residues and short flexible disordered loops are available for PTM. In disordered proteins/regions, the majority of residues are exposed and provide a readily available platform for epiproteomic modification. Disorder is particularly common in regulatory proteins such as transcription factors (TF) in both animals and plants [22]. The TF p53 has a typical platform with a number of disordered regions (often highly charged) attached to a more structured domain that interacts with DNA. The number of proteins that p53 interacts with, the roles played by epiproteome changes in p53 that link with protein–protein interactions, and the types of PTM events have grown into a very complex interacting network [23,24]. The p53 platform epitomizes the plasticity of disordered proteins and the vital importance of epiproteomic changes to their flexible response. PTM changes are clustered in and around Molecular Recognition Features (MoRFs)—short semi-ordered segments within a largely unstructured backbone that drive interactions with multiple protein partners [25] (see Figure 1).

Disorder-associated properties of p53 have likely also played a significant role in the evolution of this protein family. In a recent study of the evolution of p53 and related proteins in metazoans, Joerger et al. [26] suggest that mutations which stabilized formation of tetrameric p53 forms early in the evolution of vertebrates may have freed up the C-terminal region to adopt a disordered structure. Disorder then may have allowed the C-terminus to undergo numerous PTM and evolve the ability to interact with multiple partner regulatory proteins. They suggest that this disorder assisted evolutionary path allowed p53 to acquire many novel somatic functions by rewiring signalling pathways. Different parts of the p53 disordered regions have been shown to possess significant variation in divergence rates [27]. Indeed, it has been argued for some time that disordered regions in general have novel properties and show increased rates of mutation that suggest they are often under diversifying selection pressure and may be important to allow organisms to adapt [28,29,30,31].

The nature of the role played by RPM1-Interacting protein 4 (RIN4) in plant defence is an enigma. That its role is important is hard to question, as RIN4 is targeted directly or indirectly by a number of plant pathogen effectors [32,33]. Moreover the activity of effectors result in several different epiproteomic changes to RIN4 including phosphorylation [34,35], proteolytic cleavage [36], proline isomerisation [37] and acetylation [17]. We have suggested that, like p53, RIN4 is largely intrinsically disordered and is therefore a viable platform for multiple PTM events [32]. As for p53, RIN4 has MoRFs that correlate with conserved motifs and sites of critical importance within RIN4 that are targeted for epiproteomic modification by pathogens and/or the cell itself, or are juxtaposed to modified residues (Figure 1). Two known examples of how RIN4 works are illustrated by recent research [33,37]. Chung and colleagues suggest that two phosphorylation sites within RIN4 are in competition with each other and may be responsible for driving RIN4 in the direction of either innate (molecular pattern triggered) immunity or effector triggered immunity. The authors suggest that RIN4 is a ‘phospho-switch’ and I note that one of these sites (T166*p*-phosphorylated) sits in the middle of one of the MoRFs identified by Sun et al. [32], while the other sits near the boundary of this MoRF (S141*p*). Li and colleagues [37] identify a proline isomerisation site at P149 in the same MoRF that is, in turn, influenced by phosphorylation at T166. The T166*p* epiproteomic variant has a reduced affinity for the ROC1 enzyme that drives a *cis* to *trans* isomerisation at P149. These first examples of multiple proteomic forms illustrate how RIN4 constitutes the most compelling example yet of a plant protein playing a parallel role to that of p53, as a platform that appears primed to ‘collect’ epiproteomic signals.

### 2.4. Characteristics Required for an Integrated Response to Simultaneous Challenges

In order to be able to integrate responses, a hub must be capable of multiple interactions with various partners and collect signatures from various input pathways that can then be coherently interpreted. A high number of flexible and reversible interactions, and ability to make subtle changes to the equilibrium between the various states of control, would constitute a further advantage for such a hub. As these requirements match key characteristics of disordered regions it has been recognised for some time that such hub proteins are highly enriched for disorder [38]. Epiproteomic modification to MoRFs or neighbouring sites in a disordered platform could either block interactions, block other PTM changes at the same site (e.g., acetylation of a serine residue sometimes phosphorylated), or change the charge profile in disordered regions that then changes the dynamic of how (and/or whether) a MoRF interacts with a specific partner. While a degree of subtlety is important sometimes, a hard on-off switch will be important at other times. Disordered proteins can also undergo major conformational switches and even these can be linked to epiproteomic changes by adding larger modifying groups (e.g., glutathionylation, or AMPylation), isomerisation events around critical prolines, or by targeting the entire protein for proteolytic degradation for example.

In a recent analysis of human cells, Chavez and colleagues [39] used novel protein cross-linking methods combined with mass spectrometry to directly identify PTM decorated proteins that are physically associated with each other in complexes. New software advances have also enabled data analysis to focus on cross-linked peptides [40]. In cross-linking analyses distance constraints can be imposed by the type of chemical linker arm used, while addition of biotin groups permits enrichment for cross-linked fragments (e.g., by using avidin-mediated affinity capture technologies). Although the majority of cross-linked peptides identified were derived from homo-dimer interactions, acetylated and methylated peptides from core histone proteins participating in hetero-dimers were particularly common in this analysis. Almost half of the cross-linked histone peptides were found to contain at least one PTM event. Histones are known carry a number of highly significant PTM events. The multiple cases of linkages found between specific peptides increases the likelihood that these have biological relevance in terms of the protein interaction zones between the partners. Interestingly many of the cross-linked peptides with PTM contained modified lysine or arginine residues (residues that are also particularly enriched in disordered regions of proteins). Cross-linking sites were common in the disordered N- and C-termini of histones. In fact, it has been known for more than two decades that the histone tails are the sites where some of the most significant PTM takes place and that these modifications play key roles in the formation or dissolution of chromatin remodelling complexes. These tails serve as recognition sites for chromatin assembly as well as the assembly of the multi-component transcription machinery [41]. The largely positively charged disordered N-terminal tail also contributes to inter-nucleosome binding by contacting an acidic patch in the structured component of histone H2A/H2B dimers to influence histone stacking [42].

The cross-linking analysis allowed Chavez and colleagues [39] to build a significant interactome network map and highlights the importance of the combination of disordered regions and PTM to interactions in such networks, the hub position occupied by histones and the importance of their lysine/arginine rich disordered tails to drive their ability to organize into multi-component complexes. Other biophysical methods of experimentation can also provide indications of how closely associated proteins are in vitro or in vivo. Hydrogen-deuterium exchange (HDX) provides a measure of how exposed different parts of proteins are to PTM [43]. Changes in HDX patterns upon binding with partners can indicate likely interaction zones in protein complexes and were initially used to map antibody binding sites [44]. Other techniques like Förster (fluorescence) resonance energy transfer (FRET) also lend themselves to analysing protein disorder. For example, Vassall et al. [45] used FRET measurements to analyse the order-to-disorder transition of the myelin basic protein (MBP). MBP is largely disordered in aqueous conditions but forms alpha helical recognition fragments upon binding to membranes and its protein partners. The MBP FRET studies, when combined with other tools used to probe structural transition in largely disordered proteins (such as circular dichroism and the membrane-mimetic solvent trifluoroethanol), yielded some surprising results. The data suggested that an intermediate conformational form between disorder and alpha helical state is in fact more compact than the alpha helical form (the latter would normally be expected to have more compactness). This longer form may provide a better bridge across to its complexing protein partners as well as facilitating faster binding to the membrane. Disorder-associated characteristics possessed by histone hubs allow them to integrate epigenetic marks with downstream modifications in mRNA expression response and transfer signals between the epigenetics and transcriptomics levels of response. The disorder properties of MBP on the other hand allow MBP to peripherally attach itself to the cytoplasmic membrane as well as interact with both cytoskeletal proteins like actin and signalling proteins that respond to Ca^2+^-triggered protein cascades.

One of the ways that cells coordinate their response to changing situation such as stress is to form recognizable sub-cellular organelles. Examples include stress granules (SG), processing bodies (P-bodies) and nuclear stress bodies. Such organelles do not contain membranes, a factor that differentiates them from permanent cellular compartments like the ER, nucleus and mitochondria. Functional organelles must be able to keep interacting with their surrounding liquid environment and yet they must have an ability to form an interphase boundary with this environment. In a recent review Uversky [46] suggests that disorder can provide a crucial component required for forming this liquid-to-liquid interphase. Examples of this are the role that the RNA-binding protein TIA-1 plays to promote assembly of SG through its disordered domains and the disordered regions of a number of the RNA-binding proteins found in human and yeast stress granules. The latter were found to be able to undergo liquid-liquid phase transition in vitro on their own, or when combined with RNA [47]. The phase separated droplets promoted by this organisation can then also recruit other proteins with disordered regions. Furthermore mutations in the key disordered regions or PTM sites involved in regulation can then lead to aberrant fibers or granules that may then contribute to neurodegenerative conditions.

### 2.5. How Can Multiple Diverse Signals Be Coordinated in Real-Time?

Responses need to be organised at both the temporal and spatial levels. An important biological question is how can organisms create control points that match such elaborate requirements? Significant PTM changes can be very rapid with response times measured in minutes as opposed to hours or even days for many other types of regulation responses [48]. Rapid response makes this level of regulation ideal for responding in real time to challenges perceived by the organisms. A successful reaction to stress is dynamic and requires both sequential, temporal and spatial separation of components and the ability to be nimble in response. The high degree of sophistication required by a successful response is elegantly matched with the opportunities offered by disordered platforms to rapidly integrate PTM signals through multiple MoRFs, multiple targeted PTM sites and reversible as well as competing PTM changes at particular sites. Moreover, PTM changes can also be spatially compartmentalised by limiting where matching substrates and enzymatic functions are co-expressed. As discussed above compartmentalisation can even be aided or driven by the ability of disordered proteins to contribute to phase transition in examples like stress bodies.

Importantly many PTM changes are reversible involving balancing modifications such as phosphorylation/dephosphorylation or acetylation/de-acetylation. Pathogens in turn interfere with PTM processes by developing modifications that can compete with these changes, e.g., phospholyase reactions that break a unique phospho-threonine bond in a protein kinase activation site and make this site un-available for re-phosphorylation [15]. The very properties that make PTM changes so dynamic also make this level of response technically very demanding to illustrate. In order to capture such dynamic potential, sampling time needs to be adjusted to a much finer timescale than commonly used. In addition, techniques that can capture protein associations in real time and are not affected by their readily reversible nature (such as cross-linking techniques) will be required. This will need to be matched with detection techniques sensitive enough to identify any PTM, yet robust enough to be able to scan across complex proteomes. Physical and software enrichment strategies that can overcome the challenge of these limitations in concert with much more sensitive mass spectrometry instrumentation have recently become available. I suggest that disordered protein regions in particular have properties that indicate they are likely to feature prominently in these novel analyses in the near future. Their dynamic ability to change their binding partnerships and to be decorated by multiple PTM events, as illustrated by the examples of p53, RIN4 and histones presented above, suggest that this is one of the major reasons that disorder has become such a common feature of proteins in complex multi-cellular organisms. Indeed this fits with the proposal that disorder was a key enabler on the road to multi-cellular lifestyles [31]. The ability of disordered regions to sense the physiological milieu in which they find themselves by a combination of PTM events, charge profiles and electrostatic interactions suggests that sensing change in this milieu is a specific biological niche that disordered proteins occupy.

## 3. Conclusions

The animal and plant exemplars, p53 and RIN4, show some key similarities. I suggest that their disordered platform is specifically designed to integrate diverse signals that arrive via alternate post-translational changes inside (or sometimes in close proximity to) MoRFs. PTM changes have a great deal of flexibility and can be very rapid and reversible (e.g., phosphorylation and de-phosphorylation) and the term epiproteomics evokes the dynamic nature of these changes. In addition to reversibility, PTM sites can be; locked in competitive battles (e.g., phosphorylation and acetylation [24]), display competition between sites (as illustrated by the RIN4 phospho-switch concept [33]), result in more subtle shifts in equilibrium (e.g., by changing the charge profile and flexibility of the environment around a MoRF), or result in drastic conformational changes suited to acting as a molecular on/off switch (e.g., by proline isomerisation or multiple phosphorylations). I suggest that the main role of the p53 and RIN4 (and probably many other) proteins containing large disordered domains is to act as sensors and integrators of stress signals from multiple distinct sources via changes to the epiproteome. Moreover, this could explain why examples such as RIN4 and p53 play such important roles in plant and animal disease respectively.
